# Schlafens Can Put Viruses to Sleep

**DOI:** 10.3390/v14020442

**Published:** 2022-02-21

**Authors:** Eui Tae Kim, Matthew D. Weitzman

**Affiliations:** 1 Department of Microbiology and Immunology, Jeju National University College of Medicine, Jeju 63241, Korea; 2 Department of Biomedicine & Drug Development, Jeju National University, Jeju 63241, Korea; 3 Division of Protective Immunity and Division of Cancer Pathobiology, The Children’s Hospital of Philadelphia, Philadelphia, PA 19104, USA; weitzmanm@chop.edu; 4 Department of Pathology and Laboratory Medicine, University of Pennsylvania Perelman School of Medicine, Philadelphia, PA 19104, USA; 5 Epigenetics Institute, University of Pennsylvania Perelman School of Medicine, Philadelphia, PA 19104, USA

**Keywords:** Schlafen, SLFN, innate immunity, virus, restriction factor, immune evasion

## Abstract

The Schlafen gene family encodes for proteins involved in various biological tasks, including cell proliferation, differentiation, and T cell development. Schlafens were initially discovered in mice, and have been studied in the context of cancer biology, as well as their role in protecting cells during viral infection. This protein family provides antiviral barriers via direct and indirect effects on virus infection. Schlafens can inhibit the replication of viruses with both RNA and DNA genomes. In this review, we summarize the cellular functions and the emerging relationship between Schlafens and innate immunity. We also discuss the functions and distinctions of this emerging family of proteins as host restriction factors against viral infection. Further research into Schlafen protein function will provide insight into their mechanisms that contribute to intrinsic and innate host immunity.

## 1. Introduction

In 1998, the Schlafen (SLFN for humans; Slfn for mice) gene was first reported in the study of murine thymus development. The first Schlafens discovered were the murine genes *Slfn1–4.* When Slfn1 is expressed ectopically in NIH-3T3 fibroblasts, it induces G0/G1 cell cycle arrest; this observation led to the coining of the term “schlafen” from the German word meaning “to sleep” [1]. Later research found that Schlafens play roles in a variety of cellular functions, including anti-proliferation and cell differentiation [2,3,4,5,6,7], cancer cell migration, proliferation and invasion prevention [8,9,10,11], sensitization of cancer cells to DNA-damaging drugs [12,13,14,15,16,17], and inhibition of viral replication [18,19,20,21,22,23,24]. As studies on the Schlafen family have expanded in recent years, substantial progress has been achieved towards understanding how the proteins in this family have distinct functions. Excellent recent review articles have described their significance for the field of cancer biology [25]. The Schlafen proteins also have roles in controlling viruses and the host immune system. Here, we address the functional similarities and differences amongst Schlafen family members in terms of their roles in regulating virological and immunological features. These recent findings provide inspiration for future research directions into this emerging protein family.

## 2. Schlafen Family Members and Protein Composition

**Figure 1:**
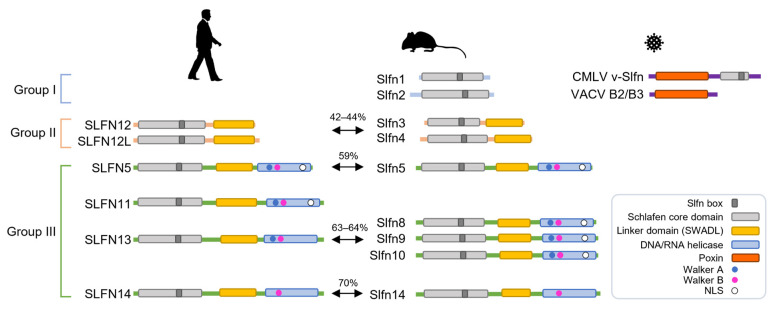
A comparison of the linear domain structures of the Schlafen family members. The domains of Schlafens in humans, mice, and viruses are depicted. The percentage of sequence identity between orthologous proteins is indicated. Walker A and Walker B motifs are located within the C-terminal helicase domain of some group III Schlafens. NLS, nuclear localization signal.

Schlafen gene family members are highly homologous across many mammalian species. Nine Schlafen proteins are expressed in mice from chromosome 11, and six have been found in humans from chromosome 17 (Figure 1) [3,26]. Despite the fact that *Slfn-like 1* (*Slfn1L*) is expressed on mouse chromosome 4, there is an opinion that it is not considered a ‘bona fide’ Schlafen family member due to the extremely low similarity to *Slfn* genes [26,27]. In addition, *Slfn6* and *Slfn7* are considered to be sequences derived from either *Slfn3* or *Slfn4* isoforms or other mouse paralogues [1,27].

Schlafen members fall into three distinct groups, each with its unique set of characteristics and functions (Figure 1). Group I has a divergent AAA ATPase-associated domain containing a common Slfn-box region that has been termed the Schlafen core domain, and is shared with the other two groups [2,28,29,30,31]. The Schlafen core domain is horseshoe-shaped and contains zinc finger motifs that are highly conserved in all members of the Schlafen family proteins. Groups II and III contain an additional linker domain following the Schlafen core domain, which harbors the SWADL motif defined by the amino acid sequence pattern S-W-(A/S)-(V/G/L)-D-(L/I/V) with unknown function [3,29]. Only group III proteins feature an extended carboxyl (C)-terminal domain that matches superfamily I of DNA/RNA helicases [2]. The Schlafen core domain lacks the Walker motif. The Walker A and B represent structural motifs for nucleotide binding, and were discovered in the AAA family of ATPases [32]. Due to the absence of Walker motifs, Schlafen proteins in groups I and II may lack ATPase activity. Putative DNA/RNA helicase domains of some group III Schlafen members have AAA domains with Walker motifs that appear to be enzymatically functional [18,33,34]. These are incomplete in murine and human Schlafen 14, which possess only the Walker B motif [31]. In addition, the C-terminal extension of some group III Schlafens possesses a nuclear localization signal (NLS) and may have nuclear functions (Figure 1) [20,24,25].

Except for the platypus, a monotreme, the Schlafen family is found in practically all mammals. Sequences similar to Schlafen genes were discovered in the amphibian *Xenopus laevis* and the fish species *Callorhincuys milii*, but not in any other non-mammalian organism. Interestingly, sequences similar to Schlafens have been found by bioinformatic analysis of genomes for orthopoxvirus (OPV), such as vaccinia, variola (smallpox), and cowpox viruses [1]. Subsequent sequencing of the camelpox virus (CMLV) identified another Schlafen-like protein called 176R. This protein consists of 502 amino acids and has a C-terminal sequence that is comparable to the Schlafen core domain of murine Schlafens. Some of these viral Schlafen (*v-Slfn*) genes retain an entire open reading frame (ORF). While the ORF for *v-Slfn* is intact in the genomes of camelpox, monkeypox, cowpox, mousepox, and taterapox viruses, protein expression is restricted in other OPVs, such as the vaccinia virus (VACV), due to ORF fragmentation [1,26,35]. Sequences of v-Slfn were found to be similar to the mouse and rat group I Schlafen, but lack the C-terminal domain. This implies that, while the progenitor virus of OPV may have acquired an intact Schlafen from rodents, the ORF was fragmented due to mutations acquired over time [26,27].

## 3. Regulated Expression of Schlafens in the Immune System

Schlafen family members have been revealed to be induced by several stimuli, including CpG-DNA [36], LPS [36,37,38], and pathogens, such as *Brucella*, *Listeria* [39], and rhinovirus [37]. Type I IFN and IFN receptors have been implicated in the induction of Schlafen genes, implying that Schlafens are IFN-stimulated genes (ISGs). In 2010, it was first reported that IFNα influences the expression of members of the Schlafen gene family [40]. The data presented in this study reveal that type I IFN is a potent inducer of numerous mouse Schlafen family members, including members of group I (*Slfn1* and *Slfn2*), group II (*Slfn3*), and group III (*Slfn5* and *Slfn8*). IFN-activated Stat proteins and p38 MAP kinase operated differently in their regulation of interferon-induced expression [41]. In Stat1 deletion in mouse embryonic fibroblasts, IFN-dependent expression of all Schlafen genes was reduced relative to parental cells, ranging from a partial reduction in *Slfn3* to total transcriptional defects in *Slfn1*, *2*, *5*, and *8*. Interestingly, *Slfn5* expression was completely independent of Stat3, but it was increased in Stat3 knockout cells. The function of p38 MAPK-activated signaling cascades is required for the complete transcriptional activation of ISGs. However, while p38 MAPK is required for IFN-dependent expression of Schlafen genes in groups I and II, interestingly, group III gene expression is not dependent on p38 MAPK. In the absence of p38 MAPK, IFN-dependent mRNA expression of *Slfn1*, *Slfn2*, and to a lesser extent *Slfn3*, was suppressed. The group III Schlafen genes, *SLFN5* and *SLFN8*, on the other hand, were induced by IFN in a p38 MAPK-independent manner [41]. Notably, neither Stat3 nor p38 MAPK was necessary for *Slfn5* induction, indicating that alternative regulatory mechanisms are involved in this process.

The induction of ISGs by type I IFNs requires the presence of interferon-stimulated response elements (ISREs) in the promoter region of the ISG, which enables transcriptional activation via the binding of the ISGF3 transcription factor, a complex of phosphorylated STAT1/STAT2 heterodimers, and IRF9 [42]. The inducibility of Schlafens by IFNα or IFN stimuli was lower than for MxA, a conventional ISG [37]. Analysis of transcription factor binding sites using the MatInspector program [43] showed that MxA has six ISRE sites, whereas most human Schlafen genes have just one canonical ISRE [37]. Although the Schlafen family belongs to the group of classical ISGs regulated by the STAT complex, some Schlafens are expressed through the noncanonical IFN pathways or undefined mechanisms. Considerable levels of Schlafens are expressed in various cells, including primary fibroblasts and malignant cancer cells, in the absence of IFN activation [18,20,44]. The sensitivity of Schlafen expression to IFN varies according to cell type. For example, SLFN5 expression is suppressed in malignant melanoma compared to normal melanocytes. IFNα stimulation, on the other hand, significantly increased *SLFN5* expression, whereas *SLFN11*, *SLFN12*, *SLFN13* were not affected [40]. In contrast, IFN stimulants, such as poly I:C and 5′ ppp-dsRNA, increased *Slfn5* expression slightly, but not significantly, in mouse macrophage RAW 264.7 cells, whereas *Slfn14* expression was significantly increased [19].

In the 5′-flanking region of the *Slfn2* gene, one copy of a putative NF-κB binding site and two copies of AP-1 binding sequences are found. It has been demonstrated that CpG-DNA and LPS treatment of macrophages requires the functional interaction of NF-κB and AP1 within the promoter element [36]. Scanning the promoter region of Slfn4 with JASPAR (jaspar.cgb.ki.se) revealed the presence of AP1 and PU. 1 binding sequences, as well as two copies of IFN response elements STAT1 and IRF1 binding sequences [38]. In addition, a Gli1 binding site also exists within the promoter. Gli1, a Hedgehog signaling effector, is required for the activation of the *Slfn4* promoter, which means that the role of Slfn4 is critical in the appearance of macrophages expressing IL1β or TNFα [45]. In cancer cells, epigenetic inhibition of gene expression via CpG promoter island hypermethylation is a frequent occurrence [46]. Several studies have reported hypermethylation of the *SLFN11* gene promoter [14,46,47,48,49]. The silencing of *SLFN11* by promoter CpG island hypermethylation is linked to a greater resistance to platinum compounds for cancer chemotherapy [14]. Hypermethylation of a CpG promoter island inactivates *SLFN11* gene expression. This methylation is catalyzed by two main DNA methyltransferases, DNMT1 and DNMT3B [14]. The fact that DNMT3B expression in monocytes is very low, or barely detectable [50], may imply that elevated levels of *SLFN11* expression in monocytes are related to hypermethylation. It is also known from germinal center B cell differentiation studies that histone modifiers, such as EZH2 and HDACs, regulate epigenetic expression of SLFN11 [48]. In addition, SLFN11 expression and the B cell lineage-specific repressor PAX5 have been shown to have a nearly perfect inverse correlation [48]. A potential PAX5 binding site (GCGTGAC) exists in the promoter region of *SLFN11*, suggesting that PAX5 may be one of the repressors of *SLFN11* in B cells.

The Schlafen members are expressed at different phases of thymocyte development and peripheral T cell activation in mice. *Slfn1* and *Slfn2* are drastically elevated during the transition from CD4 and CD8 double-positive to single-positive maturation stages. However, expression levels of both genes decrease after T cell activation [1,2,51]. *Slfn3* is highly expressed in single-positive T cells throughout thymocyte development. *Slfn3* is also expressed at a higher level in natural CD4^+^ CD25^+^ regulatory T cells than in CD4^+^ CD25^−^ cells. *Slfn3* expression is increased in CD4^+^ CD25^-^ T cells upon activation, but decreased in CD4^+^ CD25^+^ T cells following activation with anti-CD3/CD28 stimulation. TGF-β stimulation also decreases *Slfn3* expression in the CD4^+^ T cell subset, suggesting that *Slfn3* may be a novel marker of T cell activation [52]. *Slfn4* is detected early and decreased during thymocyte development, showing the opposite phenomenon to *Slfn1* [1,2]. *Slfn4* mRNA levels are upregulated during macrophage activation, whereas they are downregulated throughout differentiation. Myelopoiesis is disrupted by constitutive *Slfn4* expression in the myeloid lineage, implying that downregulation of *Slfn4* gene expression during macrophage differentiation is critical, and *Slfn4* may act as a modulator of this lineage [38]. Unlike the other groups, *Slfn5*, *8*, *9*, and *10* in group III do not change quantitatively during thymocyte development. During T cell activation, however, there was a significant downregulation of *Slfn5* and *Slfn8* expression, while *Slfn9* expression increased and *Slfn10* expression remained relatively constant [2]. Since *SLFN14* is expressed at an exceedingly low level in T cells, it is unlikely to be linked to T cell fate [37].

The human Schlafen family is also associated with immune cell proliferation and T cell maturation. With the exception of *SLFN14*, all human Schlafen proteins are expressed natively in monocytes, monocyte-derived dendritic cells (moDCs), and T cells [37]. The expression levels of *SLFN5* in T cells, and *SLFN11* in monocytes and moDCs, are notably high. The expression of *SLFN5* and *SLFN11* changes slightly throughout moDC differentiation. The expression of *SLFN12L* and *SLFN13* is relatively modest in monocytes at rest, but appears to be elevated during differentiation into moDCs, whereas *SLFN12* expression is markedly reduced [37]. Thus, the downregulation and upregulation of each Schlafen family protein may represent distinct requirements for these proteins in moDC function.

It is intriguing that there appears to be a regulatory feedback mechanism for transcriptional control within the Schlafen family [53]. The loss of *Slfn3* by knockout decreases *Slfn4*, *Slfn8*, and *Slfn9* expression in the ileal mucosa, while increasing *Slfn1* and *Slfn5*. In addition, *Slfn3* deficiency decreases *Slfn4* expression and increases *Slfn8* and *Slfn9* expression in the thymus and spleen, where immune cells mature and/or proliferate [53]. The promoters of all members of the Schlafen family contain regions for binding of the Kruppel-like factor-6 (KLF6) transcription factor. The NFAT-related factors ING4, ZNF333, and KLF4, are also predicted to bind to most Schlafen promoters. These transcription factors from the KLF family play different roles in gastrointestinal cell differentiation and proliferation, and have different expression patterns [54]. This suggests that members of the KLF and Schlafen families may have feedback loops that act as regulators of gastrointestinal and immune cell fate in different ways [53].

## 4. Immunodeficiency of Schlafen Mutants

It has been observed that the Elektra mutant is a homozygous mutation of murine *Slfn2*, and confers vulnerability to viral and bacterial infections [55]. The mortality rate of mice after murine cytomegalovirus (MCMV) infection was significantly high compared to that of the wild-type control mice [55]. In mice with the Elektra phenotype, CD8^+^ and CD4^+^ T lymphocytes fail to expand. When compared to the wild-type cells, these cells had a higher rate of apoptosis. In response to T cell activation signals, this mutation is thought to cause apoptosis [9]. Elektra mice also showed a significantly lower level of T cells in response to infection with lymphocytic choriomeningitis virus. Elektra T cells, similar to recently activated T cells, fail to maintain cellular quiescence, and enter a post-mitotic phase. T cells lose their proliferation potential and die in response to proliferation/activation signals, resulting in reduced T cell populations in the Elektra mutant mice [9].

There have been reports of a patient with a large heterozygous loss of the *SLFN11*, *SLFN12*, and *SLFN13* genes on chromosome 17 [56]. This patient was discovered to have substantial abnormalities in T cell proliferation and cell cycle regulation. Interestingly, the patient had upper thigh Merkel cell carcinoma, a kind of carcinoma associated with viral infection, and was regarded to be susceptible to cancer, having been diagnosed with T cell lymphoma. The patient’s blood and plasma had substantial Epstein–Barr virus and Torque teno virus DNA, indicating that the patient was vulnerable to viral infections. The patient had normal CD4^+^/CD8^+^ immune cell distribution and a typical distribution of naïve and memory cells, but had aberrant T cell proliferation and excessive T cell death [56].

Mutations in *SLFN14* have been linked to macrothrombocytopenia and excessive bleeding [57,58,59,60,61]. In addition, platelet function is diminished in patients with these mutations [61]. This *SLFN14* mutation presents a species-specific phenotype, with platelet abnormalities in humans and severe microcytic erythrocytosis in mice [62]. Thus, SLFN14 may be an essential player in mammalian hematopoiesis, and may play a role in determining platelet and erythroid lineage commitment in particular species. Furthermore, platelets are now known to have roles in a variety of innate and adaptive immunological responses, which goes far beyond the classic conception of platelets as only hemostatic and thrombolytic agents [63]. Therefore, it can be demonstrated that SLFN14 is profoundly implicated in immunological control through platelet formation and function regulation.

## 5. SLFN5 as an Innate Immune Signal Modulator

Although type I IFNs play an important role in host defense against pathogen infection, their production must be properly regulated to avoid inordinately harmful immune responses. Thus, negative regulators are essential for cells to recover from IFN signaling, since IFN production dysregulation leads to autoimmune disorders. Some ISGs have the ability to regulate pathways that impact their own expression, either positively or negatively. For example, ISG56 is associated with the adapter protein STING, and disrupts STING interaction with downstream molecules VISA/MAVS or TBK1, inhibiting virus-induced IRF3 activation, IFN-expression, and cellular antiviral responses. Another negative regulator is the ISG15 deconjugating protease ubiquitin-specific peptidase 18 (USP18). USP18 inhibits JAK-STAT signaling by interacting with IFNAR2 in a protease-independent manner [64]. 

Human SLFN5 has been reported to be a negative regulator of IFN-induced gene transcription [65]. It was found that STAT1 is present as a complex that binds the SLFN5 protein in a type I IFN-dependent manner, and binds to the ISRE element in the promoter of ISGs. SLFN5 appears to serve as a repressor of STAT1-induced gene transcription through direct protein interaction. Consistent with this, it was demonstrated that SLFN5 is enriched on the promoters of type I IFN-inducible ISGs, where STAT1 binds. Microarray experiments revealed that SLFN5 knockout cells expressed more ISGs than wild-type cells, suggesting a potential role for SLFN5 in regulating STAT1-mediated type I IFN-induced transcriptional activation of ISGs [65]. Similarly, in human foreskin fibroblasts and HeLa cells, basal level ISG15, a well-known antiviral protein, increased due to SLFN5 depletion; additionally, a rapid induction of ISG15 protein expression by DNA viruses, such as human cytomegalovirus (HCMV), was observed [20]. Accordingly, SLFN5 appears to be a transcriptional repressor of IFN-gene transcription, as well as an IFN-stimulated response gene. 

ZEB proteins are zinc-finger E homeobox-binding transcription factors best known for their role in driving epithelial-to-mesenchymal transition and metastasis in some cancers, including *BRCA* mutant cancer cells [66,67]. They are also widely expressed by immune cells, and regulate important transcriptional networks necessary for immune cell differentiation, maintenance, and function [68]. It has been found recently that human SLFN5 can inhibit *ZEB1* transcription by directly binding to the SLFN5 binding motif on the *ZEB1* promoter, thereby maintaining the epithelial cell morphology and inhibiting metastasis in *BRCA* mutant cancer cells [69,70]. SLFN5 increases PTEN by downregulating the transcription of *ZEB1*. Through the PTEN/PI3K/AKT/mTOR axis, an increase in PTEN inhibits lung adenocarcinoma growth and promotes apoptosis [47]. Although the SLFN5 interaction with the *ZEB1* promoter in immune cells has not been validated, these reports suggest roles for SLFN5 as a multifunctional modulator of immune cells. Interestingly, SLFN12 inhibits ZEB1; however, unlike SLFN5, it is assumed to influence post-transcriptional regulation due to its cytoplasmic localization without the nuclear localization signal sequence. SLFN12 overexpression accelerated ZEB1 proteasome degradation and slowed ZEB1 translation in triple-negative breast cancer cells [9].

## 6. SLFN5, a Double-Edged Sword in IFN Therapy

Some malignancies can be treated with IFN therapy in combination with chemotherapy and radiation. Hematological malignancies and lymphomas can be treated with this therapeutic approach [71]. Recombinant IFNα2b is given to patients with recurrences of melanomas [72]. Hepatitis B and hepatitis C are treated with IFNα and other antiviral drugs, typically combined [73,74]. The anticancer effects of type I IFNs have become extensively recognized in recent decades, particularly their involvement in mediating interactions between tumors and the immune system.

In mouse malignant melanoma and renal cell carcinoma, IFNα promotes the expression of *Slfn1*, *Slfn2*, *Slfn3*, *Slfn5*, and *Slfn8*. The loss of *Slfn2*, *Slfn4*, or *Slfn5* increased cell proliferation and anchorage-independent malignant growth, while decreasing the antiproliferative effect of IFN, implying crucial roles for Schlafens in tumorigenesis and neoplastic cell growth control [75]. All human Schlafen mRNA expression was induced in normal melanocytes by IFN therapy, while only *SLFN5* was induced in malignant melanoma cells and renal cell carcinoma cells [8,40]. When melanoma cells are stimulated with IFN, SLFN5 expression is considerably increased, decreasing cancer cell proliferation. In contrast, the depletion of *SLFN5* boosted the ability of melanomas to form colonies, even in the presence of IFN [40]. This suggests a potential role of SLFN5 in the anticancer effects of IFNα. However, SLFN5 also potentially reduces the anticancer effect of IFN in glioma cancer cells by transcriptionally co-repressing STAT1-mediated IFN responses, in contrast to its beneficial role in melanoma and renal cell carcinoma [65]. Decreasing SLFN5 leads to increased cellular susceptibility to IFN-induced antiproliferative responses in glioblastoma cells, implying that SLFN5 functions as a negative regulator of the IFN response in glioma cancer cells [65]. Thus, future therapeutic targeting of SLFN5 in malignancies may require precise analysis of other associated factors, and the design of therapeutic targeting of a particular tumor may be required for the selective targeting of SLFN5.

## 7. Functions of Viral Schlafen

The presence of intact *v-Slfn* ORFs in some OPVs suggests that it may be preserved for a critical function. Although there are few investigations into the function of v-Slfn, relatively detailed in vitro and in vivo studies on v-Slfn from CMLV have been reported. The expression of this gene was confirmed 2 h after CMLV infection, and was expressed at the early stage of infection independent of viral DNA replication [35]. In contrast to mouse Slfn1, the expression of CMLV v-Slfn does not affect the proliferation of mouse fibroblasts. This is thought to be due to the lack of similarity between the first 27 amino acids of mouse Slfn1 and v-Slfn, a region that is essential for mouse Slfn1-mediated fibroblast cell growth inhibition. When the CMLV v-Slfn protein was expressed in VACV lacking intact v-Slfn, it had no effect on recombinant virus replication or plaque morphology [35]. Additionally, intradermal infection of mice with this recombinant VACV did not affect skin lesion size [35]. However, in mice with intranasal infection, v-Slfn caused less weight loss and faster recovery compared to the control groups. At three days following in vivo infection, the viral titer was the same as in the control group, but by seven days v-Slfn-mediated attenuation was clearly observed. This suggests that v-Slfn expression does not impede viral replication, but rather accelerates viral clearance by the immune system. This is consistent with the observation that the *v-Slfn*-bearing recombinant virus was delayed in spreading to the spleen, and was more rapidly cleared from this organ. In addition, more extensive recruitment of lymphocytes into infected lung tissue was observed in the presence of v-Slfn expression, although these cells were less activated. Highly virulent viruses can quickly overwhelm their host, limiting viral transmission. The idea that v-Slfn can reduce the virulence of poxviruses, allowing the virus to spread appropriately in the host population, is compelling [35].

A novel feature of v-Slfn in poxviruses was recently discovered (Figure 1). Cyclic GMP-AMP synthase (cGAS) detects cytosolic DNA during virus infection, and induces an antiviral state. cGAS activates the stimulator of interferon genes (STING) by synthesizing a second messenger, cyclic GMP-AMP (cGAMP) [76,77,78]. With the discovery of a viral cGAMP nuclease named Poxin (poxvirus immune nuclease), the immunomodulatory potential of poxviruses was given a new perspective [6]. Recent studies have demonstrated that Poxin, which is a domain of v-Slfns, can degrade cGAMP and is required to avoid cGAS-STING activation [79,80,81]. Poxin was discovered to be a product of the VACV gene *B2R*. This gene is also known as p26 in entomopoxviruses and baculoviruses [80]. Most orthopoxviruses include a v-Slfn protein composed of two domains that have evolved from different origins. According to amino acid sequence analysis, a domain resembling the baculovirus p26 sequence is fused to the N-terminus of a v-Slfn domain similar to the murine short form Schlafen [35]; this p26-like domain is Poxin, the cGAMP nuclease. VACV, in which Poxin activity was first reported, does not retain the intact v-Slfn. The loss of Poxin resulted in a considerable reduction in VACV replication in vivo [80]. The importance of v-Slfn, which includes the Poxin domain, was studied extensively in ectromelia virus (ECTV), which causes mousepox. The Poxin domain, but not the Slfn-like domain, was sufficient to inhibit cGAS-STING signaling with cGAMP nuclease activity in a manner comparable to full-length Poxin–Schlafen-like domain fusion. This suggests that the ECTV Poxin domain preserves the full potential of v-Slfn to prevent the activation of DNA sensing via the cGAS-STING axis [79]. In several mouse infection models, the replication of ECTV lacking v-Slfn was significantly attenuated, and mice displayed a robust IFN response [79]. The Poxin–Schlafen-like domain fusion of v-Slfn is highly conserved across orthopoxviruses, such as ECTV, CMLV, and the emerging zoonotic monkeypox virus, implying the importance of cGAMP nuclease activity.

The role of the Slfn-like domain in the activation of Poxin is unclear. Poxin retained its cGAMP nuclease activity in the absence of the Slfn-like domain. Nevertheless, it remains necessary to investigate why the Slfn-like domain is conserved in many OPVs. Given the aforementioned observation that the virulence of chimeric viruses was reduced by adding the Slfn-like domain of CMLV to VACV, it is a plausible hypothesis that regulating viral virulence may contribute to creating favorable conditions for virus propagation in nature.

## 8. Schlafens as Antiviral Restriction Factors

Antiviral restriction factors are host cellular proteins that operate as a first line of defense, preventing viral replication and spread. Restriction factors recognize pathogens and interfere with specific steps in the virus infectious cycle. The unique properties of restriction factors that serve to limit viruses at early stages include constitutive expression, self-sufficient activity, and immediate action [82]. Restriction factors are occasionally increased in response to IFNs. Although many cell types constitutively express restriction factors at low levels required by cells in the absence of pathogen invasion, the effective control of a pathogen frequently requires the induction of restriction factors in response to infection [83]. Since Schlafens belong to a group of ISGs whose expression is elevated in response to viral infection or stimulation with various pathogen-associated molecular patterns (PAMPs) [36,37,38,39], it has been postulated that they may have antiviral activity.

**Figure 2:**
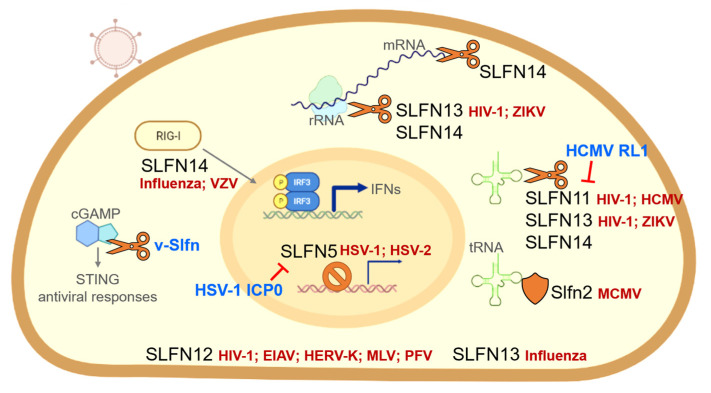
Summary of antiviral action of Schlafens. Schlafens function as intrinsic restriction factors that inhibit viral infection in a variety of ways, including (1) Slfn2, which contributes to T cell-mediated immunity by protecting tRNA from oxidative stress-induced cleavage; (2) SLFN5, which inhibits HSV gene expression; (3) SLFN11, SLFN13, and SLFN14, which have nucleolytic activity against tRNA, rRNA, and mRNA; and (4) SLFN14, which enhances RIG-I-mediated immune response. Viral immune evasion mechanisms include ubiquitin-mediated degradation of SLFN5 by HSV-1 ICP0 and SLFN11 by HCMV RL1. The STING-associated antiviral response due to cGAMP cleavage can be attenuated by v-Slfn.

Along with the discovery of the cell biological functions of Schlafens throughout the last decade, interactions with viruses have also been uncovered. In this section, we describe the known antiviral functions of Schlafens, reviewing them in the chronological order in which they were reported (Figure 2). The immune evasion mechanisms by which viruses antagonize many restriction factors have been elucidated. Furthermore, consistent with the theme that viruses can antagonize restriction factors as part of immune evasion mechanisms, there are some recently reported examples of viral strategies to counteract the antiviral action of Schlafens.

Schlafens belonging to the different groups have been reported to have distinct roles, during infection, with many viruses. There is some evidence that the malfunction of group I mouse Slfn2 predisposes cells to virus infection in terms of acquired immunity [55]. Group II SLFN12 is an antiviral factor candidate against vesicular stomatitis virus and various retroviruses, including HIV-1, equine infectious anemia virus (EIAV), human endogenous retrovirus type K (HERK-V), murine leukemia virus (MLV), and primate foamy virus (PFV) [84,85]. However, studies on the interaction of these short or intermediate forms of Schlafens with viruses are lacking, and most studies so far have focused on the antiviral function of group III Schlafens. Therefore, it is crucial to investigate whether the C-terminal extended domain of Schlafens plays a significant role in their intrinsic restriction factor function.

### 8.1. Roles of SLFN11 during Virus Infection 

Human SLFN11 was first reported in 2012 as a potent inhibitor of human immunodeficiency virus 1 (HIV-1) that interferes with viral protein production [18]. It was discovered by Li et al. that SLFN11 binds transfer RNAs (tRNAs) and suppresses protein production selectively dependent on codon usage [18]. Further research revealed that equine SLFN11 inhibits the formation of EIAV by a mechanism similar to that employed by human SLFN11 [23]. A systematic investigation of the HIV replication cycle demonstrated that SLFN11 does not affect reverse transcription, integration, or the generation and nuclear export of viral RNA, nor does it interfere with viral particle budding or release. Instead, it was found to induce the selective inhibition of viral protein synthesis. By exploiting a particular viral codon bias on the A/T nucleotide, SLFN11 functions at the moment of viral protein production. Although the antiviral effect of SLFN11 was similar to that of other viruses with an uncommon codon bias, such as influenza, it was not effective against adeno-associated virus or herpes simplex virus (HSV). These findings established that SLFN11 is a highly effective interferon-inducible restriction factor for retroviruses, such as HIV, that mediates antiviral effects via codon usage discrimination [18]. This intriguing finding may partially explain the previously observed IFN suppression of viral protein-specific synthesis in HIV-infected cells [18,86]. It also highlights how the immune system can exploit possible differences between self and non-self in order for host cells to target and eliminate viruses. There does not appear to be a preference for tRNA type in the binding of SLFN11 to tRNAs [18]. It will be necessary to conduct biochemical experiments to unveil how SLFN11 modulates tRNA function and influences virus-specific codon usage. SLFN11 is highly expressed, not only in CD4^+^ T cells, but also in monocytes and moDCs [37,87]. CD4^+^ T cells are known to be the primary reservoir for latent HIV infection, and HIV latency can also be established in monocytes and macrophages [88]. Thus, high expression of SLFN11 in these cells is thought to have a role in HIV latent infection and may be a key component of the innate immune response to HIV.

It was recently discovered that mouse Slfn2 binds to tRNA and inhibits its degradation in an oxidative stress environment [89]. Although this study showed that Slfn2 inhibited murine cytomegalovirus (MCMV) infection, the result was due to T cell-mediated adaptive immunity [89]. Nonetheless, these observations merit a thorough examination of the interaction between tRNA modulation of Slfn2 and murine retroviruses, as well as the parallels and differences with human SLFN11. Since the N-terminal portion of SLFN11 is involved in tRNA binding, there may be evolutionary similarities in sequence with the short form Slfn2. Additionally, the discovery that SLFN13 and SLFN14 participate in tRNA modulation paves the path for future investigations to identify whether Schlafens share common functions in tRNA biology [24,90].

Since the incoming viral genome of positive-sense single-stranded RNA viruses requires immediate translation to allow replication, these viruses are particularly sensitive to the effects of SLFN11 on protein synthesis. This has been demonstrated in the *Flavivirus* genus, including the West Nile virus (WNV), dengue virus (DENV), and Zika virus (ZIKV) [21]. There are similarities and differences in the mechanism of action of the Schlafen proteins against flaviviruses and lentiviruses. The N-terminal portion of SLFN11 is essential and sufficient for antiviral activity, as it prevents virus-induced alterations in the tRNA repertoire of infected cells. In contrast to WNV infection, which affected only a subset of tRNAs in SLFN11-deficient cells [21], HIV-1 raised tRNA levels overall in the absence of SLFN11 [18].

The ability of SLFN11 to regulate the abundance of tRNA pools could be related to the sensitivity of cells to DNA-damaging agents. Several studies have found that cancer cells with higher SLFN11 expression are more vulnerable to DNA-damaging agents [12,33,91,92]. Higher SLFN11 levels may limit the number of particular tRNAs that influence the translation of DNA repair proteins encoded by codon-biased open reading frames, such as ATM and ATR [93]. In addition, SLFN11 irreversibly inhibits DNA replication at DNA damage sites in a C-terminal helicase domain-dependent manner [34,94]. It has been known that various viruses exploit proteins involved in the DNA damage response of host cells for their effective replication [95]. The involvement of DNA damage control proteins ATM and ATR in HIV infection has been studied extensively. ATM has a positive effect on late gene expression of HIV and the function of Rev, a viral post-transcriptional regulator [96]; meanwhile, ATR kinase activity is required to complete the viral DNA integration process and support the survival of transduced cells [97]. In ZIKV infection, the ATM signaling pathway increases viral replication [98]. These findings suggest that Schlafens should be further investigated in terms of host cell resistance to viruses that favorably exploit DNA damage responses to ensure efficient replication.

ZIKV has generated widespread concern in recent years because of its ability to induce birth abnormalities in infants and Guillain–Barré syndrome in adults. ZIKV can be transmitted sexually, survive in the male reproductive system [99], and, in females, pass the placenta to infect the fetus [100]. Limited information is available on the effects of ZIKV on reproductive health and fertility. Given that SLFN11 is not expressed in the placenta or testes [22], additional research is needed to discover whether it is also connected with prenatal and sexually transmitted infections. 

The SLFN11 gene evolved under repeated positive selection in primates [22]. Furthermore, the antiviral efficiency of SLFN11 was highest in non-human primate species, such as gibbons and marmosets, but less effective in humans and in bonobo species that are evolutionarily close to humans, indicating that the effects of SLFN11 have become highly species-specific over time [22]. SLFN11 is functional in the absence of infection and reduces protein production from certain host transcripts [18,93]. This implies that SLFN11 may inhibit protein synthesis from non-codon optimized transcripts in general, thereby pre-establishing an unfavorable cellular environment for viral protein synthesis.

Viruses have evolved ways that counteract host restriction factors. Although a decreasing trend in SLFN11 proteins in HCMV-infected cells was demonstrated [101], viral antagonists for SLFN11 have not been discovered for many years. However, the antiviral effect of SLFN11, and its viral antagonistic mechanism on HCMV, have recently been demonstrated [102]. The late-expressed protein RL1 of HCMV targets SLFN11 for proteasome degradation, and is the first discovery of a viral antagonist to this restriction factor. In this study, it was revealed that the cellular CRL4 E3 ubiquitin ligase complex is additionally involved in the degradation of SLFN11 by RL1 [102].

In spite of the fact that SLFN11 has a significant impact on HIV, WNV, and ZIKV replication, these viruses can still replicate in cells that express SLFN11. Compared to other flaviviruses or HIV, DENV replication is significantly reduced by SLFN11 expression [21]. This suggests that DENV is more susceptible than other viruses to the effects of SLFN11. Thus, it would be expected that DENV lacks an antagonistic mechanism for SLFN11, whereas WNV, ZIKV, and HIV-1 may possess veiled antagonistic mechanisms.

The mechanism by which the phosphorylation of SLFN11 by protein phosphatase 1 catalytic subunit ɣ (PPP1CC) regulates type II tRNA cleavage ability has been reported [103]. Cellular protein activity is well known to be regulated by viral kinases [104]. No evidence has yet been found to support the hypothesis that viruses regulate the phosphorylation of SLFN11 through virus-encoded kinases, or indirectly through host cell kinases, such as PPP1CC. Further research is required to explore the possibility that viruses exploit protein phosphorylation to circumvent the antiviral activity of Schlafens, as has been observed for other host restriction factors [105,106,107,108,109,110,111,112].

### 8.2. Roles of SLFN13 during Virus Infection 

Crystallographic analysis revealed that SLFN13 is a new class of tRNA/rRNA nucleases [24]. In addition, it was also reported that SLFN13 had an antiviral function against HIV and ZIKV by inhibiting protein synthesis through nucleolytic activity, similar to SLFN11. However, the key determinant of tRNA cleavage by SLFN13, which blocks protein synthesis, is the secondary structure of the tRNA and is not correlated with the anti-codon sequence [24], which appears to be different from the codon usage-based mechanism of SLFN11. The sequence of the N-terminal domain of SLFN13, which is required for enzyme function, is conserved in other Schlafen proteins. However, specific positively charged amino acid residues are different. It was confirmed that certain family members, such as human SLFN5 and mouse Slfn1, are not involved in tRNA cleavage [24]. Thus, it is likely that the distribution of positively charged amino acid residues inside the N-terminal domain can determine the ability and selection tendencies of tRNA/rRNA cleavage as well as antiviral spectra for other Schlafens.

Influenza A (PR8) and B (Victoria) virus infections were observed to induce *SLFN13* mRNA expression in human lung adenocarcinoma A549 cells [19]. This induction was more robust in viral *NS1*-deficient mutant infection, presumably due to the ability of NS1 to repress RIG-I-mediated activation of the *IFN* promoter [113]. Furthermore, depletion of SLFN13 increased influenza A and B virus plaque development, implying that SLFN13 promotes antiviral responses to these viruses [19]. However, whether the SLFN13 antiviral function against influenza virus is related to tRNA/rRNA cleavage is unknown. Therefore, there is a need to determine whether Schlafen nucleolytic activity is a common mechanism for Schlafen-mediated antiviral function. The absence of an antiviral effect of SLFN11 against a virus with a negative-sense single-stranded RNA genome [21] suggests the existence of a mechanism independent of the anti-influenza virus function of SLFN13.

### 8.3. Roles of SLFN14 during Virus Infection 

Antiviral functions have also been reported for SLFN14, and expression is increased by influenza A infection [19]. Depletion of SLFN14 limited the upregulation of IP-10, a major ISG, following influenza infection. These results suggest a possible mechanism by which SLFN14 recognizes the viral RNA genome, enhances the activating RIG-I mediated signal and inhibits influenza replication [19]. However, it is necessary to confirm whether SLFN14, same as for helicases, such as DDX1 or RIG-I, truly detects the viral genome [114]. SLFN14 delays the nuclear translocation of nucleoprotein NP. Delayed nuclear translocation of NP may impair viral replication by impairing viral ribonucleoprotein nuclear transport.

In addition to its effects on RNA viruses, SLFN14 has also been demonstrated to possess antiviral activity against DNA viruses, such as the varicella-zoster virus (VZV). VZV infection induces SLFN14 expression and inhibits viral antigen production in cells overexpressing SLFN14 [19]. Although SLFN14’s antiviral mechanism against RNA viruses and DNA viruses is assumed to be distinct, additional research on the putative helicase domain of SLFN14 and the association of RIG-I-mediated IFN signaling is required for a more detailed mechanism analysis. In addition, since cell types expressing SLFN14 are very limited, or the expression level is low [115], the genuine function of SLFN14 in virus-infected cells remains to be evaluated.

SLFN14 has been found to have ribosome-associated endonuclease activity, and can degrade tRNA, rRNA, and mRNA [90]. There is no sequence specificity or preferred structure specificity in RNA cleavage, and this enzymatic activity is strictly Mg^2+^- and Mn^2+^-dependent and ATP-independent [90]. However, only the C-terminally truncated short version of SLFN14 exhibited enzymatic activity, whereas full-length SLFN14 lacked endonuclease activity and did not bind to ribosomes [90]. This feature appears to be a way to maintain the integrity of cellular RNAs. Since the SLFN14 protein is present at low levels in most cells and occurs in the nucleus, an inactive precursor state similar to caspase may shield cellular RNAs against non-specific endonuclease activity. Viral infections induce SLFN14 expression in a manner similar to that of RNase L [116], and it may participate in the clearance of total cellular RNA to inhibit viral reproduction. However, it is still required to demonstrate that SLFN14 is processed into the active form after infection or in certain environments.

### 8.4. Roles of SLFN5 during Virus Infection 

In human cells, SLFN5, along with SLFN11, is the most abundant Schlafen family protein [18]. SLFN5 is a nuclear member of the Schlafen family, which has been linked to immune cell proliferation and differentiation [28,117].

Studies report that influenza virus, WNV, and rhinovirus infection result in increased SLFN5 expression [37,118,119]. However, the function of SLFN5 against these viruses has not been investigated, and unlike SLFN11, it has been experimentally established that SLFN5 has no antiviral activity against HIV infection [18]. A recent investigation of SLFN5 revealed an antiviral action and mechanism against HSV-1, a virus with a double-stranded DNA genome [20]. In that study, host factors associated with HSV-1 DNA were isolated using a proteomics technique called Isolation of Proteins On Nascent DNA (iPOND), which identifies proteins accumulating on newly synthesized DNA [120,121,122]. When applied to HSV-1 infection with wild-type and mutant viruses, this technique revealed that SLFN5 undergoes proteasomal degradation as a result of accelerated ubiquitination by viral protein ICP0.

The HSV-1 immediate-early protein ICP0 facilitates viral gene transcription and virus reactivation from latency. ICP0 features a ubiquitin E3 ubiquitin ligase domain that antagonizes host defenses through the proteasomal degradation of intrinsic antiviral host factors [123,124]. The HSV-1 DNA has been found associated with a number of ICP0 degradation targets, which were also shown to inhibit the production of viral genes and/or the activation of antiviral cell signals [124]. Although previous studies identified ICP0 substrates as restriction factors, the mechanism for suppressing viral gene expression is not fully understood. In this recent study, it was confirmed biochemically that ICP0 specifically ubiquitinated and degraded SLFN5 via the proteasome [20]. The direct interaction between ICP0 and SLFN5 was found to occur via the extended C-terminal domain of SLFN5, a region that is absent in SLFN11, which was not targeted for degradation. The C-terminal region of SLFN5 contains an intrinsically disordered region, a frequent feature of cellular proteins bound by viral factors, such as ICP0 [125]. The antiviral effect of SLFN5 on HSV-1 is more clearly observed in mutant viruses lacking the E3 ligase activity of ICP0 than in wild-type viruses. The observations that HSV-1 targets SLFN5 [20] and HCMV targets SLFN11 [102] suggest that proteasome-mediated degradation may be a more common viral strategy used to antagonize Schlafen restriction. 

The antiviral mechanism of SLFN5 has been proposed to bind to viral DNA and inhibit RNA polymerase II loading onto viral gene promoters [20]. Additionally, it was proven that, unlike other Schlafens, it had no effect on mRNA degradation. Although putative helicase activity may be the mechanism by which SLFN5 represses viral DNA gene transcription, the Walker A helicase motif of SLFN5 does not affect its antiviral function [20]. SLFN5 does not appear to have any DNA sequence specificity. The examination of SLFN5 binding to promoter and gene body regions revealed no apparent preference; however, there was a noticeable tendency to bind more to viral DNA over cellular DNA [20]. A recent study of SLFN5 structure demonstrated a high affinity for double-stranded DNA, and identified the residues involved in nucleic acid binding [31]. Although SLFN5 binds tRNA, it does not share the endoribonuclease activity reported for other Schlafens [31]. SLFN5 was also shown to have a preference for binding to free DNA over nucleosome-bound DNA [31]. Perhaps the ease of access to euchromatic viral DNA in the lytic infection environment may contribute to selective binding to viral genomic DNA [126].

**Figure 3:**
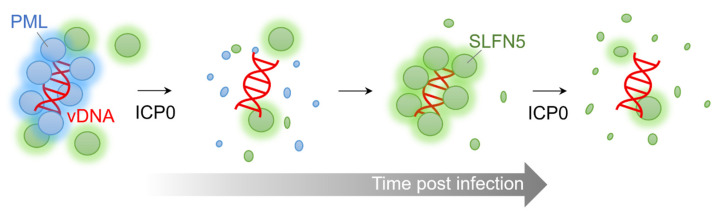
A cooperative inhibition model of SLFN5 against viral genomes. Immediately after viral infection, the PML protein captures HSV-1 DNA and suppresses viral gene expression. Subsequently, PML is rapidly degraded and removed by ICP0, and the SLFN5 protein, in turn, recaptures HSV-1 DNA and suppresses viral gene transcription. This second line of inhibition is also counteracted by ICP0-induced degradation.

The interaction between SLFN5 and viral DNA was detected for both incoming viral genomes and actively replicating viral genomes at viral replication compartments [20]. The host PML protein, a well-known HSV-1 restriction factor, as well as an ICP0 substrate, also accesses incoming viral DNA and inhibits viral gene transcription [127,128,129,130]. Although further biochemical studies are required, the ICP0-mediated degradation of SLFN5 appears to be less efficient than PML degradation [20]. Immediately upon infection, the majority of HSV-1 DNA is surrounded by PML protein; however, when ICP0 is expressed, PML is rapidly eliminated, and viral DNA is once again entrapped by SLFN5 protein [20]. This suggests that PML and SLFN5 may work cooperatively to create an unfavorable environment for viral gene expression. Therefore, the role of SLFN5 may be a second line of defense supporting the antiviral function of PML (Figure 3). The observation that SLFN5 regulates immune responses, and is also targeted by ICP0, suggests that it may form part of a ‘self-guarded’ immune pathway to monitor infection. The degradation of SLFN5 by ICP0 could thus trigger the activation of secondary immune responses. This guard hypothesis was recently suggested for MORC3, another target of the ICP0 [131]. Further studies are required to understand the connections between SLFN5 and other regulators of HSV-1 infection.

A recent analysis of the HSV-1 single-cell transcriptome revealed that β-catenin recruitment to the viral replication compartment is required for HSV-1 gene expression [132]. SLFN5 is known to inhibit cell migration and proliferation by inhibiting the expression of β-catenin [133,134], implying that SLFN5 could also indirectly affect HSV-1 gene expression.

Despite the fact that SLFN5 has no antiviral activity against retroviruses, it does have an antiviral effect against HSV-2, an *alphaherpesvirinae* close to HSV-1. Interestingly, the results for *betaherpesvirinae* HCMV differed depending on the stage of infection [20]. Within the first 24 h after infection, SLFN5 depletion promotes the expression of viral immediate-early and early gene transcripts; however, this is reversed in the late phase, resulting in the reduced expression of these viral genes in the absence of SLFN5. As a result, HCMV replication yield is slightly decreased in SLFN5-deficient cells. One difference between HSV-1 and HCMV is the time course of infection, with HSV-1 replication being much faster than HCMV. Since SLFN5 inhibits STAT1-mediated ISGs transcription [65], SLFN5 depletion may result in increased ISG signaling, which decreases HCMV replication. Indeed, knocking down SLFN5 resulted in higher levels of ISG15 expression, which increased further following HCMV infection [20]. As a result, SLFN5 is directly involved in reducing early viral gene expression, and it appears to have a distinct effect on HSV-1 at later stages. Another DNA virus, adenovirus, was unaffected by SLFN5, and viral infection did not result in SLFN5 protein degradation [20]. Together, these data suggest specificity to the antiviral activity across the Schlafen protein family, similar to what has been observed for other families of host restriction factors.

## 9. Conclusions and Future Perspectives

Continuous and in-depth research on the Schlafen family has made significant progress toward elucidating the roles of Schlafen proteins in recent years. Current studies have shown that Schlafen proteins play critical roles in regulating both the immune response and the cell cycle. Some of these proteins are associated with tumor treatment susceptibility and drug resistance [13,14,15,135]; thus, the biological function of Schlafen family proteins in tumor cells provides new methods and ideas for tumor detection and treatment. In addition, the Schlafen proteins exhibit a relatively broad inhibitory effect on retroviruses via RNA modulation to inhibit translation. Schlafen proteins have also been implicated in viral infection indirectly via interferon signaling. The discovery of a mechanism for the direct inhibition of viral gene expression through SLFN5 binding to viral DNA in the nucleus has highlighted the potential diversity in the antiviral mechanisms of the Schlafen family.

Numerous findings to date demonstrate that the Schlafen family has a role in a variety of cellular responses, including immune cell development and intrinsic/innate immunity. This protein family is, unquestionably, an important target for cancer treatment, as well as research into understanding and preventing viral infections. However, functional studies on the Schlafen proteins are still in their infancy, and there are many important questions that remain to be solved. Although the Schlafen family shares several similar domains, they show functional differences. These distinctions imply that the Schlafen family members confer specificity to their antiviral activities, highlighting the importance of studying structural properties and functional mechanisms. Fortunately, structures have been determined for rat Slfn13 [24] and human SLFN5 [31], providing insight for ongoing studies of Schlafen family proteins. Furthermore, the limitations of animal experiments for clinical application must be overcome. The Schlafen family showed a rapid evolutionary tendency in several rodents, and the degree of conservation between rodent and human Schlafen genes is not high. For example, SLFN5 and SLFN11 are the most abundant and highly studied in various cells, but the similarity between mouse and human of SLFN5 is only 59% based on the amino acid sequence identity, and there is no SLFN11 ortholog in mice. SLFN5 and SLFN14 are the only orthologs shared between mice and humans (Figure 1). Therefore, there is a need to develop a new platform, such as organoid models, that can substitute for in vivo studies.

Given the diverse functions of Schlafen family proteins, various binding partner proteins in the cell are expected to play roles in their regulation. Although no report on the results of a global proteomic approach to interactome has been published, it is critical to discover and study the role of binding partners as factors that differentiate the function and regulation of intracellular activity of Schlafen family proteins. The study of associations between Schlafen expression level and cancer prognosis can be applied to virus-mediated tumor research or treatment using viral vectors. Despite their name, the Schlafen field is far from a sleepy one. Ongoing studies will provide important insight into both virus and tumor biology, and will suggest ways that their unique activities can be harnessed for therapeutic applications.
